# Bussuquara Virus: A Neglected Orthoflavivirus with Broad Distribution Across Central and South America and the Caribbean

**DOI:** 10.3390/v17020183

**Published:** 2025-01-27

**Authors:** Madeline R. Steck, Michaela Buenemann, Nikos Vasilakis

**Affiliations:** 1 Department of Pathology, University of Texas Medical Branch, Galveston, TX 77555-0610, USA; mrsteck@utmb.edu; 2 Department of Geography and Environmental Studies, New Mexico State University, Las Cruces, NM 88003, USA; elabuen@nmsu.edu; 3 Institute for Human Infections and Immunity, University of Texas Medical Branch, Galveston, TX 77555-0610, USA; 4 Center for Vector Borne and Zoonotic Diseases, University of Texas Medical Branch, Galveston, TX 77555-0609, USA

**Keywords:** *orthoflavivirus*, mosquito-borne virus, transmission cycles, clinical manifestations

## Abstract

Bussuquara virus (BSQV) was first discovered in the Brazilian Amazon in 1956. It is an arthropod-borne virus (arbovirus) in the genus *Orthoflavivirus*, family *Flaviviridae*. Since its discovery, BSQV has been sporadically detected across the South (Brazil, Columbia, and Argentina) and Central (Panama and Mexico) America and the Caribbean (Grenada), but there is minimal BSQV surveillance due to limited public health awareness and a lack of specific or sensitive diagnostics. BSQV exposure has been reported in a wide range of host and vector species, including humans. Little information is available in the literature and herein we summarize the published historical findings on BSQV and suggest a pathway for future studies to better understand its potential emergence into human populations.

## 1. Introduction

Bussuquara virus (BSQV) was identified as a novel species in 1956, through a surveillance program managed by the Belém Virus Laboratory (BVL) of Instituto Evandro Chagas, in the Amazon rainforest near Belém, Pará State, Brazil [1,2]. BSQV was first isolated from the blood of a sentinel monkey (*Aloutta belzebul*), with later isolations from rodents and mosquitoes sampled in nearby surveillance sites. Early BSQV characterization involved studies of vertebrate host susceptibility, vector competence, and vector–host transmission [3].

**Figure 1:**
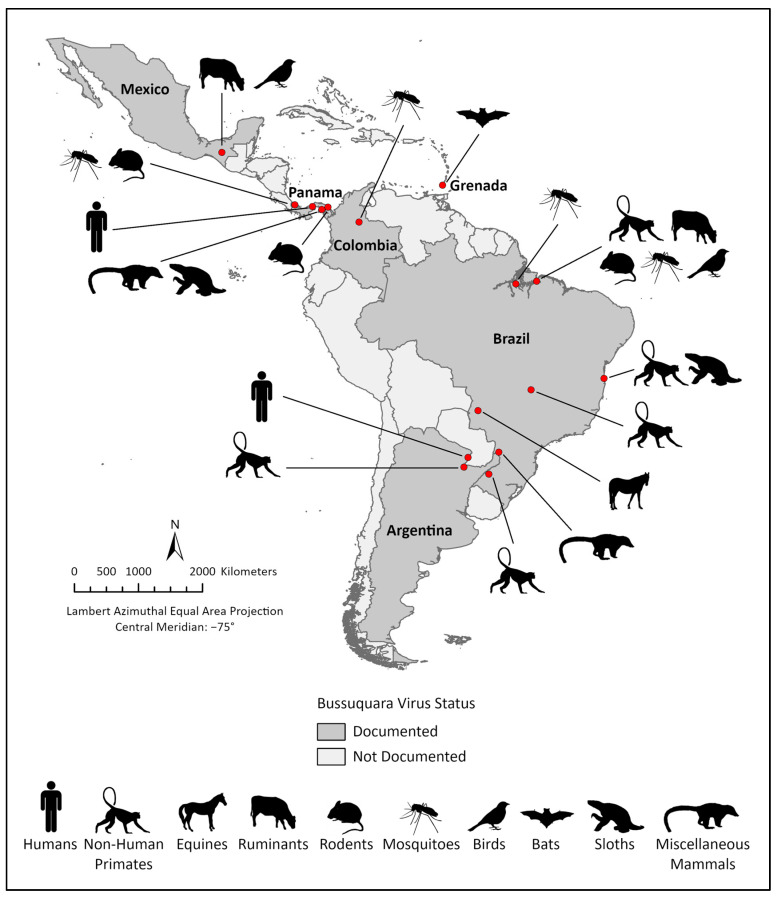
**BSQV distribution in the Americas**. Geographic range and epidemiological landscape of BSQV. Hosts from which BSQV and/or antibody have been identified are indicated by representative graphics [Figure created with ArcGIS Pro3.4].

Unfortunately, most of the seminal BSQV investigations are inaccessible with limited reproducibility due to a lack of published methods or raw data [3]. Few field surveillance studies today include BSQV and the last serological evidence of human exposure was documented over two decades ago [4]. BSQV has been sporadically detected in various vertebrate and mosquito species across Central and South America and the Caribbean suggesting transmission across a broad vector–host range (Figure 1), whereas its transmission cycle(s) remains unclear, as does the emergence potential for an urban transmission cycle. The only verified reports of human exposure have been reported in Panama [5] and Argentina [4]; however, no BSQV diagnostics are commercially available. Historically, early arbovirus diagnosis and the discovery of novel arboviruses relied on serological testing, such as hemagglutination inhibition (HI), complement fixation (CF), or *in vivo* neutralization by intracranial inoculation in mice (NT) [6]; the plaque reduction neutralization test (PRNT) has since been established as a contemporary *in vitro* NT alternative [7]. Most orthoflavivirus infections can be inapparent, while diagnosis in symptomatic patients is often confounded by serological cross-reactivity and undifferentiable febrile symptoms [8]. Clinical triage in arbovirus endemic regions is focused on the most prominent arboviral threats which limits the detection of less common pathogens. For example, in Brazil and surrounding south American countries, the public health focus is towards chikungunya (CHIKV), Zika (ZIKV), and dengue (DENV) viruses [9,10,11]. Other circulating viruses of concern include Rocio (ROCV), yellow fever (YFV), West Nile (WNV), Western and Venezuelan equine encephalitis (WEEV and VEEV, respectively), Madariaga (MADV), Mayaro (MAYV), and Oropouche (OROV) viruses [8,12,13,14,15], but their targeted surveillance is atypical outside of an ongoing outbreak.

Zoonotic arbovirus spillover and emergence from sylvatic, epizootic, and urban environments is an increasing threat worldwide [16,17,18,19,20]. Such events, fueled by uncontrolled urbanization, global trade, deforestation, and climate change, may lead to localized outbreaks or epidemics in human/animal populations, as witnessed with the emergence of ZIKV, SARS-CoV-2, and OROV [21,22,23]. To facilitate accurate clinical diagnosis and elicit a coordinated public health response during future emergence scenarios, it is pertinent to proactively characterize viral species of high emergence potential. We consider that the BSQV literature reviewed here provides a premise for investigating the potential risk and consequences of BSQV emergence in the Americas.

## 2. History, Classification, and Taxonomy

BSQV was identified as a novel virus using NT assays in suckling mice against a subset of representative orthoflaviviruses, such as Ilheus virus (ILHV), DENV-1, ZIKV, YFV) [1]. However, BSQV was not placed into a serocomplex for several decades, with antigenic solitude maintained until 1974, when a de Paola and colleagues’ cross-neutralization study with the forty-two recognized “Group B” (e.g., orthoflavivirus) species placed it in the Aroa serocomplex [24]. The Aroa serocomplex (*Orthoflavivirus aroaense*) also includes Aroa (AROAV) (isolated 1972, Venezuela, sentinel hamster), Iguape (IGUV) (isolated 1973, Brazil, sentinel swiss mice) [25,26], and Naranjal (NJLV) (isolated 1976, Ecuador, sentinel hamster) [27] viruses. It would be advantageous to characterize the basic biology and disease ecology of BSQV to its most closely related viruses. However, there is not sufficient ecological or experimental characterization for NJLV, IGUV, and AROAV, as well as for viruses in the neighboring Kokobera serocomplex [28,29]. BSQV is phylogenetically intermediate to othroflavivirus subclades with either *Culex* or *Aedes* as the principal vector genus, and it is designated as a *Culex*-vectored virus [30,31,32]. Understanding the biology and ecology of these viruses will guide targeted OneHealth surveillance, disease prevention, and control countermeasures.

## 3. Genome and *In Vitro* Characterization

To date, only one complete BSQV genome sequence, the BeAn 4073 prototype strain [1], is available in the NCBI GenBank (AY632536.4/NC_009026.2). It exhibits the standard orthoflavivirus genome organization with a positive single-strand RNA of approximately 11.8 kb in length flanked by 5′ and 3′ non-coding regions (NCRs), and contains one open reading frame (ORF) encoding three structural (capsid, pr/M, and envelope) and seven non-structural (NS1, NS2A, NS2B, NS3, NS4A, NS4B, and NS5) genes [8]. The genome is not fully characterized except for the identification of the polyprotein cleavage sites and a predicted 5′ UTR secondary structure [33]. A handful of strain isolates are named in the literature [34,35,36], with solely the prototype strain used to inform orthoflavivirus phylogenies and determine evolutionary relationships [31,37,38].

BSQV BeAn 4073 infection produces high viral titers [5–7.0 log_10_ plaque forming units (pfu)/mL] and/or cytopathic effects (CPE) in vertebrate cell cultures including chick embryo, Pekin duck kidney, and rhesus monkey kidney primary cell lines, as well as the established HeLa (human cervical cancer), BHK-21 (baby hamster kidney), Vero (green African monkey kidney), MA-104 (rhesus monkey kidney), and LLC-MK2 (rhesus monkey kidney) cell lines [39,40,41,42]. Bergold et al. characterized two BSQV strains (BeAn 4073 and CoAr 41922) in several rodent and non-human primate (NHP) cell lines and observed varied plaque morphology and viral titers [41].

Barros et al. used transmission and scanning electron microscopy to visualize morphological changes in BSQV-infected peritoneal C57Bl/6 mouse macrophages [43]. BSQV induced similar CPE both within the cell (e.g., vesicle formation, cytoplasm vacuolization, and hypertrophic rough endoplasmic reticulum) and on the cell surface (increased cytoplasmic projections) compared to YFV-, ROCV-, and St. Louis encephalitis (SLEV) virus-infected macrophages. BSQV virion size was not conclusive with an estimated range of 50–70 nm. The antiviral response [cytokines (IL-1β, TNF-α, TGF-β, and IFN-α) and nitric oxide (NO)] to the same four viruses was quantified, and each induced macrophage production of IFN-α and NO, but BSQV displayed opposite trends in cytokine inhibition (TGF-β) and exacerbation (IL-1β) [44].

These data indicate that BSQV fits several canonical features of orthoflaviviruses including genome size and organization, virion size, and replication mechanisms. Overall, there is minimal comparison to related orthoflaviviruses and a single *in vitro* comparison between the available strains. This offers an opportunity to characterize the BSQV genomes and define similarities or divergence across key motifs, particularly those associated with orthoflavivirus replication and virulence [2,45,46]. BSQV infection kinetics are undefined in vertebrate and invertebrate cells, which are major limitations in our basic understanding of species susceptibility and viral fitness in potential host and vector species. The observed deviations in cell-mediated immune responses should also be explored further to support future *in vivo* models. Such a knowledge base would support the development and testing of (i) specific and sensitive diagnostic assays and (ii) clinical countermeasures (e.g., anti-viral targets and immune therapeutics) to enable rapid identification and patient care during BSQV emergence.

## 4. Ecology, Transmission Cycles and Epidemiology

The BSQV vector–host transmission cycle remains unclear due to historically sporadic BSQV detection (Table 1). Araújo et al. used sequencing to directly identify BSQV genome fragments [47], while other studies have relied on serologic assays to detect and differentiate BSQV exposure from that of other orthoflaviviruses. BSQV has been isolated from a sentinel monkey [1], sentinel mice [48,49] and wild rodents [5,34,48,49,50,51,52], mosquitoes [35,48,49,53], and one human [5]. BSQV antibodies were also detected in a wide vertebrate species range including humans, ruminants, birds, mammals, non-human primates, and marsupials (Table 1). The primary sylvatic mosquito vector(s) species remains unknown, although isolation from mosquitoes in the genera *Culex* [35,47,48,49,53], *Trichoprosopon* [53], and *Mansonia* [49] has been reported. ArboCat lists additional isolates from *Culex (Mel) taeniopus*, *Culex vomifer*, and *Coquillettidia venezuelensis* [3], likely credited to the BVL during their 1954–1964 field surveillance studies [48,49]. A recent institutional report stated that from 1954 to 2022, BVL researchers made one hundred and nine isolations of BSQV in mice (n = 30), wild rodents (n = 45), mosquitoes (n = 28), and non-human primates (n = 1) [54].

Many instances of BSQV detection are due to arbovirus surveillance efforts led by the BVL in Brazil [48], and by the Gorgas Memorial Laboratory in the Republic of Panama [53]. The initial BSQV isolation was from a sentinel monkey caged 4–5 m above ground level in a secondary growth forest on an abandoned cocoa plantation [1,3,50]. In another nearby forest, a BVL mammal recapture program [34,49,50,51] sampled wild *Proechimys* rodents from a watershed plot surrounded by forest swamps and water canals. BSQV seroconversion was observed in 20 animals (70% of population) over a period of six months. Antigen response measured by HI assay had a long duration [until end of study (up to 425 days)] with authors postulating that antibodies might persist for the animals’ lifetime. This high level of exposure was also reported by the BVL in samples collected across multiple wild rodent populations, including *Nectomys* [24%], *Prochiemys* [70%], and *Oryzomys* [15%] [49]. Notably, *Nectomys* and *Oryzomys* are considered semi-aquatic rodent families [55]. BSQV antibodies were also detected by the BVL in one non-speciated bird, alongside isolation of virus from *Culicine* and *Mansonia* species mosquitoes [49,56]. All BSQV detection in Brazil during the mid–late 1900s occurred within the same contiguous stretch of forest proximate to the northern city of Belém, Pará State [48].

BSQV was not reported again in Brazil until the 21st century, when antibodies were detected in non-human primates [57,58,59], sloths [59], and coatis [60], alongside agricultural/domesticated animals such as water buffaloes [61], horses [62], and birds [63]. Out of eighty-nine domestic pigeons from a municipal park in Belém, five birds had monotypic activity, and one bird had heterotypic reactivity by HI to BSQV and ILHV. Notably, these birds were negative to all assayed orthoflaviviruses via NT [63], suggesting either cross-reactivity or transient immunity. Two heterotypic and four monotypic HI titers for BSQV were reported in wild water buffaloes from an unidentified location within Pará State [61]. A decade-long surveillance project in the Bahia Atlantic Forest tracked arbovirus circulation in free-ranging and semi-captive animals using HI and PRNT assays [59]. Five *Leontopithecus chrysomelas* tamarins were reactive by HI, one of which had monotypic neutralizing antibodies, while one recaptured sloth (*B. torquatus*) had seroconversion to multiple arboviruses (BSQV, DENV-2, DENV-3, ILHV, Utinga, and Caraparu) over the study period. Subsequently, BSQV genome fragments were detected by polymerase chain reaction (PCR) assay in a pool of *Culex (Mel.) portesi* collected at the Caxiuanã National Forest in northern Brazil [47] (Table 1).

In Goiânia City, within central Brazil, one *Cebus libidinosus* had monotypic HI antibodies against BSQV while another *C. libidinosus* and *Alouatta caraya* exhibited heterotypic exposure profiles to BSQV, MAYV, OROV, DENV-1,-3-4, and Capicacore (CPCV) or BSQV, OROV, and CPCV, respectively [58]. These NHPs were housed in a wild animal triage center that mostly received animals from the city’s urban parks. In southern Brazil, a vertebrate sampling study in Iguaçu National Park detected heterotypic HI antibodies in three coatis that reacted to BSQV, ILHV, SLEV, DENV1-4, NJLV, and/or YFV [64]. In Rio Grande do Sul State, primates in forested city areas were sampled three times over a year [65]; one *A. caraya* had a heterotypic response by PRNT to BSQV, CPCV, and YFV, and two others had sub-threshold BSQV neutralization titers at either first capture or recapture. In the Pantanal, a subtropical flood plain, twelve equines on cattle ranches had heterotypic BSQV neutralizing antibodies using a panel that included WNV, SLEV, ROCV, ILHV, DENV1-4, YFV, IGUV, and CPCV [62] (Table 1).

Outside of Brazil, BSQV was isolated from a *Culex* spp. pool collected at a river valley bottom within a rainforest in San Vicente de Chucuri, Colombia [35]. BSQV was next isolated from sentinel mice and *Culex* and *Trichoprosopon* mosquitoes on a cacao plantation and in an intermediate swamp forest/tropical rainforest in northwestern Panama [53]. The mosquito collections were simultaneously performed on the ground and in the canopy, with mouse traps hung right above the ground [48]. BSQV antibodies were detected in wild rodents sampled in a tropical forest in eastern Panama near to the Colombian border [52,66]. NT assays also distinguished BSQV in multiple sloths and one agouti from the tropical forests of central Panama [36]; however, the NT titers for other orthoflaviviruses (SLEV, ILHV and/or YFV) were higher than those for BSQV in each animal, suggesting an anamnestic response to prior orthoflavivirus exposure. Srihongse et al. reported the first human case in Panama in serum samples collected during the mid-1960s in a rural lake region of central Panama [5]. Interestingly, serum samples collected from volunteers residing in nearby villages revealed that approximately 6% of the participants (n = 1715) had HI antibodies predominant to BSQV and monotypic antibodies by NT in 46% (n = 383) of the HI-positive subset, suggesting active BSQV circulation in the area. A serological study in 1998 by Glowacki et al. reported BSQV neutralizing antibodies in military recruits in Argentina residing near the border with Paraguay [4], described as a region of dense forests, rivers, and non-human primates including *Alouatta* spp. [4]. More recently, Morales et al. also detected BSQV seroconversions in free-ranging *A. caraya* [57] in the same region of Argentina, suggesting active foci of BSQV transmission.

The northernmost BSQV exposure was documented by Ulloa et al. in 2003, during sampling of village households within a tropical rainforest region in southern Mexico [67]. In this study, four young cattle and one chicken had weak monotypic reactions to BSQV by PRNT, and no evidence of exposure to other endemic orthoflaviviruses (e.g., SLEV, WNV, ROCV, and ILHV). Stone et al. also reported BSQV detection in the Caribbean island of Grenada during a 2015 bat capture study, where a single bat collected near to an inhabited town had monotypic BSQV PRNT_80_ titers [68].

The ecological niche of BSQV is thus still not fully understood. The accumulated evidence to date suggests BSQV may be a generalist arbovirus that opportunistically infects a broad range of vertebrate and mosquito species. In most surveillance reports, the number of BSQV positive animals compared to the total animals sampled is low (Table 1). It is possible that either the reservoir species are not being sampled or BSQV has low baseline circulation. Other important considerations include the ecology of the sampling sites (e.g., biome) and the sampling design (e.g., horizontal across ecotones and vertical ground-to-canopy collections). These are details that help clarify the following: (i) the risk of virus spillover and spillback; and (ii) the community composition and stratification, transmission dynamics, and feeding behavior of potential vectors of transmission. The proximity of multiple BSQV detection sites to agricultural lands and urban human communities is of high interest. An understanding of BSQV bridge vectors or hosts would further indicate the risk of virus spillover from sylvatic to peridomestic/urban settings, and spillback in reverse. Documented BSQV exposure does not confirm a vertebrate species as either a reservoir and amplification host or a dead-end host, and currently there is an absence of experimental animal and transmission studies to validate such assertions.

**Figure 2:**
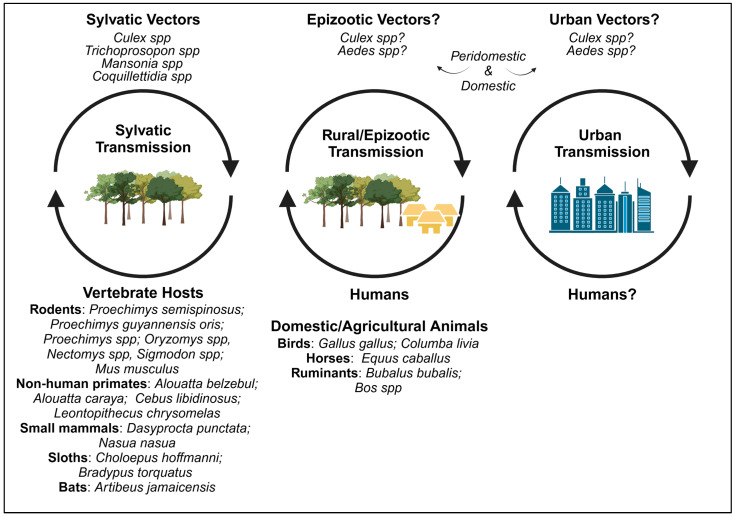
**Possible BSQV transmission cycles.** Common name and/or genus of species with positive BSQV detection [Figure made at Biorender.com].

Field isolations and phylogenetic placement suggest the principal sylvatic vectors are *Culex* spp. (Table 1), whereas the surveillance studies reviewed above imply that BSQV is maintained in an enzootic transmission cycle in tropical forests near aquatic biomes. Forest-dwelling rodents and/or birds are the likely reservoir hosts, with spillover potential into vertebrates in agricultural and peridomestic settings (Figure 2). The wide geographic range of BSQV (Figure 1) may be due to migratory birds akin to WNV [69] and SLEV [70]. Therefore, comprehensive OneHealth surveillance studies coupled with the development of animal models of pathogenesis and transmission and vector-competence studies are urgently needed.

**Table 1:** **Serologic and molecular detection of BSQV and evidence of circulation.** Evidence of current or previous infection of BSQV in multiple species across Central and South America and the Caribbean. Serologic assays included hemagglutination inhibition (HI), complement fixation (CF), and *in vivo* neutralization (NT) by intracranial inoculation in mice or *in vitro* plaque reducing neutralization test (PRNT). Genomic detection was through the reverse transcription polymerase chain reaction (RT-PCR) assay and Sanger sequencing.

Species	Common Name	Country	City	Publication Year	Method	Assay	Detection Target	No. Positive Samples	Ref.
*Alouatta belzebul*	Red-handed howler	Brazil	Belém	1959	Serology	HI, CF, NT	Virus	1	[1]
*Sigmodon* spp.	Cotton rat	Panama	NR	NR	Serology	NR	Virus	NR	[5]
*Culex* spp.	Mosquito	Colombia	San Vincente de Chicuri	1961	Serology	HI, CF, NT	Virus	1 pool (95)	[35]
*Mus musculus*	Swiss mice	Brazil	Belém	1961–1967	Serology	HI, CF, NT	Virus	9	[48,49]
*Culex (Melanoconium)* spp.	Mosquito	Brazil	Belém	1961–1967	Serology	HI, CF, NT	Virus	2 pools (51, NR)	[48,49]
*Proechimys* spp.	Rodent	Brazil	Belém	1961–1967	Serology	HI, CF, NT	Virus	15	[48,49]
*Proechimys guyannensis oris*	Guyenne spiny rat	Brazil	Belém	1963–1967	Serology	HI	Virus	1	[34,50,51]
*Proechimys guyannensis oris*	Guyenne spiny rat	Brazil	Belém	1963–1967	Serology	HI	Antibody	20	[34,50,51]
*Nectomys* spp.	Rodent	Brazil	Belém	1967	Serology	HI	Antibody	NR	[49]
*Oryzomys* spp.	Rodent	Brazil	Belém	1967	Serology	HI	Antibody	NR	[49]
*Culex* spp.	Mosquito	Brazil	Belém	1967	Serology	HI, CF, NT †	Virus	6	[49]
*Culex B1* *	Mosquito	Brazil	Belém	1967	Serology	HI, CF, NT †	Virus	1 pool (NR)	[49]
*Culex B7* *	Mosquito	Brazil	Belém	1967	Serology	HI, CF, NT †	Virus	1 pool (NR)	[49]
*Culex (Melanoconium) taeniorhyncus*	Mosquito	Brazil	Belém	1967	Serology	HI, CF, NT †	Virus	1 pool (NR)	[49]
*Mansonia tittilans*	Mosquito	Brazil	Belém	1967	Serology	HI, CF, NT	Virus	1 pool (NR)	[49]
*Mansonia venezuelensis*	Mosquito	Brazil	Belém	1967	Serology	HI, CF, NT †	Virus	1 pool (NR)	[49]
*---*	Bird	Brazil	Belém	1963	Serology	HI, NT †	Antibody	1	[49,56]
*Mus musculus*	Swiss mice	Panama	Almirante	1966	Serology	HI, CF	Virus	2	[53]
*Trichoprosopon* spp.	Mosquito	Panama	Almirante	1966	Serology	HI, CF	Virus	1 pool (NR)	[53]
*Culex cyrbda*	Mosquito	Panama	Almirante	1966	Serology	HI, CF	Virus	1 pool (NR)	[53]
*Homo sapiens*	Human	Panama	Arenosa	1971	Serology	HI, CF, NT	Virus	1	[5]
*Homo sapiens*	Human	Panama	Arenosa	1971	Serology	HI, NT	Antibody	116–176	[5]
*Proechimys semispinosus*	Tome’s spiny rat	Panama	Sasardi	1974	Serology	HI	Virus	1	[52]
*Choloepus hoffmanni*	Hoffmann’s two-toed sloth	Panama	Maje	1983	Serology	PRNT †	Antibody	6	[36]
*Dasyprocta punctata*	Central American agouti	Panama	Maje	1983	Serology	PRNT †	Antibody	1	[36]
*Homo sapiens*	Human	Argentina	Formosa Province	1998	Serology	HI, PRNT	Antibody	1–2	[4]
*Gallus gallus*	Chicken	Mexico	Chiapas Province	2003	Serology	PRNT †	Antibody	4	[67]
*Bos* spp.	Cow	Mexico	Chiapas Province	2003	Serology	PRNT †	Antibody	1	[67]
*Bubalus bubalis*	Water buffalo	Brazil	Pará State	2014	Serology	HI	Antibody	2–4	[71]
*Equus caballus*	Horse	Brazil	Pantanal	2014	Serology	PRNT †	Antibody	12	[62]
*Alouatta caraya*	Black howler	Brazil	Goiânia	2015	Serology	HI †	Antibody	1	[58]
*Cebus libidinosus*	Black-striped capuchin	Brazil	Goiânia	2015	Serology	HI †	Antibody	2	[58]
*Alouatta caraya*	Black howler	Argentina	Corrientes Province	2017	Serology	PRNT †	Antibody	1–10	[57]
*Columba livia*	Rock dove	Brazil	Belém	2017	Serology	HI, NT †	Antibody	5–6	[63]
*Leontopithecus chrysomelas*	Golden-headed lion tamarin	Brazil	Atlantic Forest, Bahia	2018	Serology	HI, NT	Antibody	5	[59]
*Bradypus torquatus*	Maned sloth	Brazil	Atlantic Forest, Bahia	2018	Serology	HI, NT	Antibody	1	[59]
*Artibeus jamaicensis*	Jamaican fruit bat	Grenada	St. David Parish	2018	Serology	PRNT †	Antibody	1	[68]
*Culex (Mel.) portesi*	Mosquito	Brazil	Caxiuanã National Forest	2019	Sequencing	NA	Virus	1 pool (NR)	[47]
*Alouatta caraya*	Black howler	Brazil	Santo Antônio das Missões	2019	Serology	HI, NT †	Antibody	3	[65]
*Nasua nasua*	South American coati	Brazil	Iguaçu National Park, Brazilian Atlantic Forest	2023	Serology	HI †	Antibody	3	[60]

† Heterotypic arbovirus exposure and/or orthoflavivirus reactivity in BSQV-positive samples; * B1 and B7 refer to BVL codes for taxonomically difficult Cx species to identify. Abbreviations: NR—not reported; NA—not applicable.

## 5. Clinical Presentation of Infection in Humans

To date, there has been one documented human BSQV case, reported by Srihongse et al. [5]. A 29-year-old Panamanian male, residing in a village 30 km northwest of Panama City, displayed symptoms of low-grade fever (continuous frontal headache, chills, and profuse sweating) and arthralgia without evidence of inflammation. Symptoms persisted for four days, and the virus was isolated from blood serum collected on the first day of illness. Serologic assays (HI, CF, and NT) indicated that the virus was antigenically similar, if not identical, to the second prototype BSQV strain isolated in Brazil, BeAn 4116. Notably, this patient had increased anti-BSQV titers detectable one year later, as well as serological reactivity to other unspecified orthoflaviviruses, albeit at a reduced potency in comparison to BSQV titers, which suggests subsequent heterotypic infection with other orthoflaviviruses. The authors described in parallel the first BSQV seroprevalence study addressed in Section 4 (Ecology, Transmission Cycles, and Epidemiology). Critically, no clinical presentations were described by Glowacki et al. for the BSQV-seropositive humans in Argentina [4].

## 6. Animal Models and Pathogenesis

Pathogenesis details from the sentinel howler (*Alouatta belzebul*) monkey that BSQV was initially isolated from are sparse [1]. The animal died nineteen days after the first blood collection, and histopathology findings (icteric serum and hepatic lesions) were indicative of yellow fever. Subsequently, only the liver was submitted for post-mortem analysis and no results of the analysis were presented.

In the BSQV animal model by de Paola et al. [72], two-day old hamsters were intraperitoneally (i.p.) inoculated with 1000 pfu of a mouse-adapted BeAn 4116 strain (11 passages in suckling mouse brains (SMBs)) and all animals succumbed five days post-infection. Organs from necropsied animals were used to determine BSQV titers or sectioned for immunofluorescence and H&E staining. BSQV was localized in liver macrophages and lymph node macrophages starting at day one post-infection, with progressive expansion through the entire brain including the cerebral cortex, cerebellum, choroid plexus, gyrus hippocampus, basal nuclei, and ependyma starting at day three, followed by the detection of neuronal necrosis at day four post-infection. Viral titers were also detected in the kidney and lymph nodes. The data suggested that macrophages played a key role in BSQV maintenance and dissemination to the CNS, which corroborated the BSQV susceptibility of murine peritoneal macrophages [43,44]. The authors of the study also stated that this neonatal hamster model matched the outcomes (e.g., succumbing to CNS disease) of an unpublished BSQV infant mouse model. Similar results were reported by Gomes and Causey, where an SMB-passaged BSQV BeAn 4073 strain inoculated intracerebrally (i.c.) in newborn or adult mice resulted in average survival times of four to six days, respectively [1].

A later study by the BVL reported the average survival time in adult mice to be 6.3 days by i.c., but i.p. inoculation was non-fatal [48]. In infant mice, survival with BSQV was shorter at 4.8 (i.c.) and 5.0 days (i.p.). Adult hamsters infected by subcutaneous (s.c.) or i.c. inoculations survived to three weeks post-inoculation and seroconverted with high antibody titers [73,74]. More recently, Amarilla et al. examined the cross-protective abilities of various orthoflaviviruses to ROCV infection [75]. Six-week-old C57BL/6 mice were i.p. inoculated with 6.0 log_10_ pfu BSQV, and the authors reported the following: (i) an absence of neurological signs with robust seroconversion three weeks post-inoculation; and (ii) upon challenge with 7.0 log_10_ pfu ROCV, mild protection (reduced mortality, body weight loss, and cumulative disease score) was observed.

Collectively, both wild and laboratory rodents, birds, and non-human primates are susceptible to BSQV infection, but its pathogenesis outcomes and mechanisms are unclear. The rodent models suggest neurotropism with age-dependent susceptibility and survival [3,72,75], while the sentinel monkey succumbed with significant liver pathology [1]. Tissue tropisms are also undefined due to inaccessible study methodology [3], insufficient histological analysis [1,75], and limited assessment of tropisms, especially neurotropism [72]. Rodents are instrumental surrogates to investigate arbovirus infection and disease mechanisms [76]; however; one human case report [5] may not comprehensively define the spectrum of outcomes of human BSQV infection. Instead of endeavoring towards specific disease phenotypes, we determined to focus future animal models towards experimental parameters (inoculation route, age, rodent species, and immune status) that recapitulate natural virus–mosquito–host interactions. A representative infection model approach is critical to clarify the sparse and contrasting BSQV tropisms and pathogenesis described in the literature.

## 7. Vector Competence

The ArboCat profile [3] states perfunctory results from vector-competence studies including (i) BSQV dissemination to *Ae. aegypti* and *Cx. quinquefasciatus* salivary glands post-intrathoracic inoculation with serially passaged (host or vector species unknown) virus, and (ii) BSQV transmission to laboratory mice using wild-caught *Culex* mosquitoes. Tesh et al. later reported evidence of transovarian BSQV transmission in *Aedes albopictus* [77]. Females were intrathoracically injected with BSQV and eggs were collected over several rounds of oviposition. Progeny were reared to adult mosquitoes, pooled, and titrated in Vero cells, with positive infection affirmed by both CPE and CF testing. Transovarian transmission was positive in at least two mosquitoes yielding a minimal filial infection rate of 1:1762 (n = 3523 divided in pools of 100 mosquitoes).

The capacity for an urban BSQV transmission cycle is unknown. Evidence of BSQV exposure in humans and domestic animals suggests spillover into epizootic cycles. Detection in NHPs and birds living within urban city boundaries (e.g., urban parks and animal conservatories) further implies that this virus may maintain silent circulation near humans. Infection in urban *Aedes* or *Culex* mosquitoes has not yet been reported, and thus historical experiments with representative species must be refined and repeated to resolve issues of (i) inaccessible methodology, (ii) inoculation route (of a potentially adapted strain) that bypasses midgut barriers, and (iii) failure to assess transmission beyond the salivary gland escape barrier. Peridomestic and domestic *Aedes* and *Culex* species have been paramount to the rapid worldwide geographic expansion, emergence, and the high human morbidity and mortality burden of multiple arboviruses, e.g., CHIKV, ZIKV, DENV, WNV, Japanese encephalitis virus (JEV), and SLEV [17,78]. It is pertinent that vector competence for BSQV in these critical species be assessed, e.g., infection (midgut), dissemination (legs and wings), transmission potential (saliva), or viremia measurements after blood-feeding on naive hosts. These data would inform vectorial capacity analyses and thus bolster risk predictions of BSQV emergence and maintenance in urban transmission cycles.

## 8. Diagnosis and Prevention

Classical serological assays distinguish BSQV exposure when a comprehensive orthoflavivirus panel is used [21,40,41,78,79]. However, there is no current incentive to surveil for BSQV, which is reinforced by the lack of specific and sensitive BSQV assays. BSQV is not included in clinical triage applications nor contemporary arbovirus surveillance studies within wildlife or human populations. The only evidence of BSQV inclusion in orthoflavivirus serological panels are the studies outlined in Table 1 and in equine arbovirus surveillance studies (all BSQV-negative) conducted in Mexico [79], Colombia [80], and Brazil [71,81,82]. BSQV is otherwise utilized to evaluate the diagnostic specificity and sensitivity of serologic [83,84], molecular [85,86] assays for other orthoflaviviruses, or the sensitivity of pan-orthoflavivirus assays [87,88,89,90]. BSQV-specific primers have been published [13,91,92] but never validated with field or clinical samples.

A confounding factor for BSQV diagnosis is that most arbovirus infections present with nondifferential clinical symptoms and are often misdiagnosed as dengue or malaria. Thus, the prevalence of human exposure in endemic regions is unknown and there are no targeted guidelines to reduce BSQV infection risk. No licensed antiviral treatments exist for orthoflaviviruses, and only a handful of them (e.g., YFV, DENV, JEV, and tick-borne encephalitis virus) have licensed vaccines available. Vector control and personal preventative countermeasures (e.g., skin-covering outfits, mosquito repellant, window and door screens) are currently the most apparent methods to avoid BSQV exposure and minimize the risk for infection. However, targeted vector control for BSQV is unlikely because the mosquito species involved in transmission remain unknown.

The re-emergence of endemic and emergence of novel orthoflaviviruses, in tandem with globalization, climate change, and uncontrolled urbanization, provides greater opportunities for individuals to be exposed to multiple orthoflaviviruses over a lifetime. This hinders accurate differentiation of past or current infection due to increased opportunities for cross-protection or cross-reactivity, respectively. It is crucial to develop BSQV-specific diagnostics for the early detection of spillover, emergence, and outbreaks. Sporadic detection is not conclusive evidence of an inconsequential impact on human health, historically or in the future. Technological advancements (e.g., sequencing, nucleic acid detection, and enzyme-linked immunoassays) since BSQV’s discovery permit the development of accurate detection assays and thus facilitate the reassessment of human exposure and disease frequency.

## 9. Conclusions and Future Directions

Overall, BSQV has a broad geographic distribution and vector–host range, and it can infect humans with significant disease outcomes. The impact of these observations is hampered by the lack of specific BSQV diagnostics, historical reliance on serologic assays, and limited animal studies. Serologic data demonstrate previous BSQV spillover events into peridomestic/domestic animals and humans. The observed proximity of BSQV-exposed vertebrates and mosquitoes to urban environments with no evidence of urban transmission warrants closer investigation to better understand the risk of BSQV emergence. To date, there is limited information on the manifestations of BSQV infections in humans and animals; however, the observations of neurotropic and/or viscerotropic disease outcomes warrant comprehensive studies in animal models to better understand the pathogenesis of BSQV infections in humans. Thus, we suggest focusing on several research avenues to understand these risks and consequences of BSQV emergence in the Americas. These include the following: (i) comprehensive characterization of all available isolates; (ii) viral fitness studies to assess BSQV’s status as a generalist arbovirus; (iii) vector-competence studies in urban and peri-urban mosquitoes from a broad geographic range; (iv) development of an appropriate animal model to assess tissue tropisms and pathogenesis; and (v) development of specific and sensitive BSQV diagnostics.

## Data Availability

Not applicable.
